# Preparation of Transdermal Gel Containing l-Theanine for the Potential Treatment of Premenstrual Syndrome: A Preclinical Study

**DOI:** 10.1089/whr.2023.0108

**Published:** 2024-02-29

**Authors:** Kaana Ando, Ikumi Sugiyama, Yasuyuki Sadzuka

**Affiliations:** ^1^ Division of Pharmaceutical and Health Sciences, Graduate School of Pharmaceutical Science, Iwate Medical University, Yahaba-cho, Shiwa-gun, Iwate, Japan.; ^2^ Department of Advanced Pharmaceutics, School of Pharmacy, Iwate Medical University, Yahaba-cho, Shiwa-gun, Iwate, Japan.

**Keywords:** premenstrual syndrome, transdermal, lyogel, l-theanine

## Abstract

**Background:**

Premenstrual syndrome (PMS) is experienced by many women who suffer from either its psychological or physical symptoms. Current treatment is limited to symptomatic therapy or oral contraceptives. On the other hand, l-theanine, which has a relaxant effect, has been reported to be useful for PMS, but its short half-life when administered orally makes it less effective. Permeability and properties of transdermal gel containing l-theanine were evaluated as a preclinical study of PMS symptoms relief formulation.

**Materials and Methods:**

Lyogel composed of stearic acid, stearyl alcohol, and propylene glycol was selected. The ratio of these components and the preparation method were investigated. Permeation of Strat-M membranes was evaluated by using Franz cells (*in vitro*). Moreover, lyogel was applied to institute of cancer research mice's backs for 10 days to examine the permeability of l-theanine.

**Results:**

l-Theanine solution did not permeate the Strat-M membrane at all in the permeation study, but lyogel allowed l-theanine to permeate. When the composition of lyogel was 4.4:11.1:296 (mmol) for stearic acid, stearyl alcohol, and propylene glycol, l-theanine absorption through Strat-M membrane was better. In skin permeation study using mice, l-theanine was detected in the serum, that is, it was proven that l-theanine penetrated the skin.

**Conclusion:**

The preparation of transdermal gels contained l-theanine was investigated as a preclinical study. The skin permeability of semisolid formulations of hydrophobic ointments, hydrophilic ointments, oily creams, creams, and lyogel containing theanine was compared and found that lyogel was the best. The composition of lyogel was also studied to obtain a formulation with good application comfort. Although it is suggested that this lyogel could be tested in clinical studies to determine whether it is effective for relief of PMS symptoms, lyogel may be suitable as an easy-to-use l-theanine-containing formulation for women that can relieve PMS symptoms.

## Introduction

Premenstrual syndrome (PMS) is a physical and psychological symptom that also develops during the menstrual cycle's luteal phase and abates within a few days of menstrual onset.^1^ The PMS symptoms are varied and divided into two main categories: physical symptoms such as abdominal bloating, breast pain, and headaches and psychological symptoms such as anger, depression, and loss of interest.^1,2^ It is reported that 95% of women have PMS and ∼5% of women fall into the premenstrual dysphoric disorder (PMDD) category, which is considered a severe condition.^2,3^

PMS and PMDD are factors that contribute to decreased ability to perform daily activities and lead to worsening of relationships.^4,5^ PMS causes have been variously attributed to estrogen; progesterone's effects on neurotransmitters, such as serotonin, opioids, catecholamines, and γ-amino butyric acid; the phenomenon of cortisol; adrenal dysfunction; vitamin B_6_ deficiency; increased prolactin levels; and insulin resistance.^6,7^ However, these are not yet fully understood.

In most cases, PMS is treated with pharmacotherapy, which includes selective serotonin reuptake inhibitors, anxiolytics, gonadotropin-releasing hormone agonists, diuretic agents, nonsteroidal anti-inflammatory drugs, and oral contraceptives.^1,8^ Clinical studies also suggested that combining pharmacotherapy with nonpharmacological therapies, such as aerobic exercise, reflexology, and yoga, may be beneficial for PMS.^8^ However, such treatment methods are symptomatic PMS treatment, and drug treatment may expose these women to the risk of developing side effects. Currently, the challenge is that many women suffer from PMS symptoms, yet no effective treatment has been established.

Green tea is a familiar beverage for Japanese people that in recent years it has gained worldwide recognition. Drinking green tea does not only rehydrate the body but is also expected to have a relaxing,^9^ antiviral,^10^ and cancer treatment effect.^11^ l-Theanine is an amino acid unique to green tea or black tea and is an l-glutamic acid ethyl amide derivative that has attracted attention for its various effects, including suppressing elevated blood pressure^12^ and preventing bladder dysfunction.^13^ In addition, l-theanine can pass through the blood‒brain barrier^14^ and is thought to cause relaxation by increasing alpha waves in the brain at 8–13 Hz.^15^

This effect suggests that oral administration of l-theanine may be effective in decreasing PMS symptoms.^16^ However, l-theanine's half-life in blood is ∼1 hour,^17^ and it is quickly metabolized and excreted after oral administration. Thus, a strategy to enhance its effect is needed. Drug delivery and *in vivo* blood half-life can be controlled by the drug delivery system. The transdermal route is one of the drug delivery systems. Transdermal drugs absorption can avoid hepatic first-pass effects, reduce administration frequency, and prolong drug efficacy.

Furthermore, it is an easy-to-use dosage form, evidenced by the fact that it can be administered to unconscious patients and patients who have difficulty swallowing.^18^ Taking advantage of the formulation's sustained release technology characteristic, transdermal formulations are also being used clinically for angina pectoris and dementia.^19^ l-Theanine in transdermal therapy was hypothesized to have a long-lasting relaxing effect on PMS by gradually reaching the systemic circulatory blood flow through the skin.

Formulations applied to the skin include transdermal patches, tapes, and semisolid formulations such as ointments, creams, and gels. As indicated, transdermal patches are generally used when systemic effects are expected. However, our aim is to develop a formulation to relieve PMS symptoms and is intended for use by women. We considered that it would be unsightly for women to apply the patch and that it would be an undesirable formulation if they were concerned about their appearance. In addition, it is commonly known as a disadvantage of patches that they can cause skin diseases such as skin rashes.

However, semisolid preparations such as ointments and creams are known to have excellent moisturizing effects and are less likely to cause skin disorders. We considered that the semisolid formulation is more suitable for use by menstruating women, who are more prone to skin problems, than a transdermal patch.

For these reasons, a semisolid formulation was selected as the formulation in this study.

This study aimed to develop a skin-permeable formulation containing l-theanine for PMS symptom relief. In this study, we focused on lyogel, a semisolid formulation for skin application, and we evaluated gelation of l-theanine, physical properties test of the formulations, and skin permeability of l-theanine as a basic study.

## Materials and Methods

### Materials

Taiyo Kagaku Co., Ltd. (Mie, Japan) gifted the l-theanine. Stearic acid and propylene glycol were purchased from FUJIFILM Wako Pure Chemical Corp. (Osaka, Japan). Stearyl alcohol was purchased from Nacalai Tesque, INC. (Kyoto, Japan). All other chemicals were commercial products of reagent grade.

### Animals

The institutional animal care and use committee of Iwate Medical University approved the animal experiment. Male Institute of Cancer Research (ICR) mice (6 weeks old, 23–25 g) were purchased from Japan SLC Inc. (Hamamatsu, Japan). The animals were given a standard experimental diet and water and maintained under controlled temperature (23 ± 1°C) and humidity (55 ± 5%).

### Determination of l-theanine

l-Theanine was dissolved in distilled water to obtain concentrations of l-theanine ranging from 0 to 0.2 mg/mL, and it was measured using a high-performance liquid chromatography (HPLC) system equipped with a ultraviolet-vis detector (Shimadzu SPD-10AVvp; Shimadzu Co., Kyoto, Japan) using acetonitrile–100 mM NaH_2_PO_4_・2H_2_O (pH4.5) (50:50, v/v). The kinetex EVO C18 100Å (5 mm, 150 × 4.6 mm) (Phenomenex Inc., Torrance, CA, USA) column had a flow rate of 0.5 mL/min and was maintained at 40°C. The detector wavelength was set at 210 nm.

### Preparation of semisolid formulations

Hydrophobic ointments, hydrophilic ointments, creams, oily creams, and lyogel were selected as the base for the transdermal semisolid formulations containing l-theanine. The formulations except lyogel were mixed and dissolved with 10% l-theanine in the base substrate at 60°C. Stirring was continuous until room temperature was reached. The detailed base materials selected were as follows: plastibase (Taisho Pharmaceutical Co., Ltd., Tokyo, Japan) for hydrophobic ointments, macrogol ointment solbase (Yoshindo Inc., Toyama, Japan) for hydrophilic ointments, hydrophobic cream (Nikko Pharmaceutical Co., Ltd., Gifu, Japan) for creams, and absorptive cream (Nikko Pharmaceutical Co., Ltd.) for oily creams.

Lyogel was prepared by referring to the preparation method of Matsushita,^20^ and the gel prepared using this method was used as the standard. The standard sample was composed of stearic acid, stearyl alcohol, and propylene glycol (4.4:23.1:230 (mmol)). l-Theanine was mixed after stearyl alcohol was dissolved at 90°C. Separately, stearic acid and propylene glycol were mixed and dissolved at 83°C, and then stearyl alcohol with l-theanine was added gradually. After mixing thoroughly, the heating was stopped. The mixture was kept stirred until it reached room temperature.

The standard formulation was modified and l-theanine-containing gel-A, gel-B, gel-C, and gel-D were prepared as shown in Table 1. Gel-A, gel-B, and gel-C were dissolved in stearic acid, stearyl alcohol, and propylene glycol together at 83°C, and then l-theanine was mixed. As soon as l-theanine was dissolved, the heating was stopped and stirring was continued until room temperature was reached. Standard and gel-B have the same composition but different gel preparation methods.

**Table 1.** Brief List of Lyogels Prepared

Group	Stearic acid (mmol)	Stearyl alcohol (mmol)	Propylene glycol (mmol)
l-Theanine predissolved in stearyl alcohol			
Standard	4.4	23.1	230
l-Theanine is mixed with all components from the outset			
A	4.4	23.1	184
B	4.4	23.1	230
C	4.4	23.1	296
D	4.4	11.1	296

Standard was mixed with the other components after dissolving l-theanine in stearyl alcohol, in contrast, gel-B is dissolved in l-theanine along with all the constituents. Gel-D was prepared by mixing l-theanine with stearic acid, stearyl alcohol, and propylene glycol, and dissolving all together at 500 rpm using a magnetic stirrer set at 83°C. After 1 hour, heating was stopped, and the mixture was stirred and then cooled to room temperature.

### Permeability study of l-theanine from formulations

Franz cells were used for l-theanine permeability studies. Strat-M membrane (Merck &Co., New Jersey, USA) with a thickness of 300 μm and a diameter of 47 mm, which is commonly used in alternative transdermal diffusion studies, was used. The receiver side was filled with phosphate-buffered saline (pH 7.8) and stirred and maintained at 34°C by circulating water. l-Theanine solution or semisolid formulations with l-theanine was applied on the Strat-M membrane (application area was 3.14 cm^2^), and the receiver solution was collected from the sampling port at a scheduled time. The l-theanine concentration in the sampling solution was determined in the same way as described in the previous section.

### Spreadability test

A spread meter (Kumagai Riki Kogyo Co., Ltd., Tokyo, Japan) was used to determine the spreadability of lyogel. After the sample was filled into a 0.5 cm^3^ indentation, the glass plate was dropped. The diameter (cm) of the circle spreads was measured 10 s after the glass plate fell and recorded as comparative values. This test was conducted at 24 ± 2°C.

### Permeability study by lyogel application on mouse skin

ICR mice were randomly divided into the l-theanine solution (*n* = 3) and lyogel (*n* = 3) groups, and their backs were shaved. Gel-D containing 20% l-theanine was applied to the back of mice (0.1 g l-theanine/animal). Application was once a day for 10 consecutive days. l-Theanine solution was diluted to 0.3 g/mL and applied to the back in the same manner as the lyogel. At 24 hours after the last application, blood was collected from their heart. The collected blood was centrifuged, and the plasma was separated. After protein removal in the plasma, the amount of l-theanine was measured using HPLC, and l-theanine concentration was calculated using a calibration curve. HPLC measurements were performed under the same conditions as in previous section.

### Statistical analysis

Data are expressed as the mean and standard error of the mean of multiple determinations. The significance of the differences in the mean values of the two groups was evaluated using Student's unpaired *t*-test. IBM SPSS Statistics Version 22 (IBM Corp., Armonk, NY, USA) was used to perform statistical analyses.

## Results

### l-Theanine permeation in semisolid formulation

**FIG. 1.**
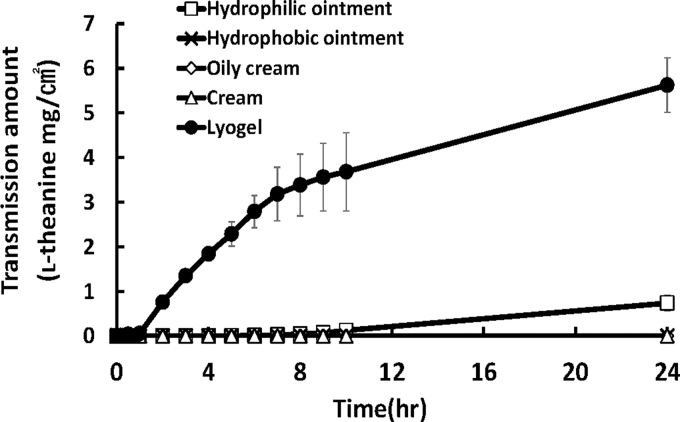
Permeability of l-theanine in semisolid preparations through Strat-M membrane. Each point represents mean ± SD (*n* = 3). SD, standard deviation.

The permeability level of l-theanine was compared with that of the semisolid formulations, which are hydrophilic ointment, hydrophobic ointment, oily cream, and cream, and lyogel formulation. It was shown that l-theanine level in lyogel was the highest permeability, with permeability at 35% after 24 hours. l-Theanine in hydrophobic ointments, cream, and oily creams hardly permeated the Strat-M membrane. l-Theanine in the hydrophilic ointments permeated the membrane, but the amount was 7.6 times higher in the lyogel formulation (Fig. 1).

### Lyogel formulation comparison in l-theanine permeation rate

**FIG. 2.**
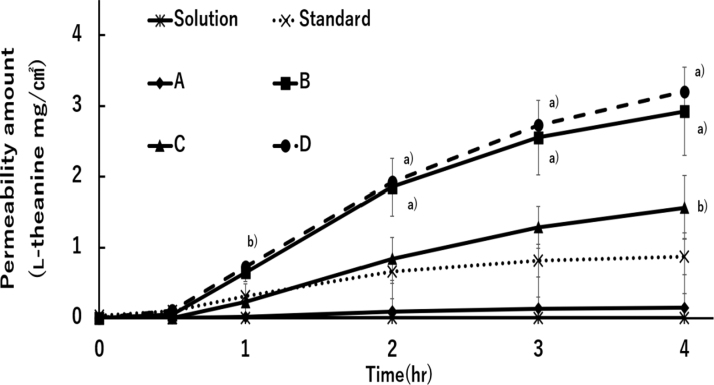
Permeability of l-theanine from 20% (w/w) l-theanine in gel-standard, gel-A, gel-B, gel-C, and gel-D through Strat-M membrane. Each point represents mean ± SD (*n* = 3–7). Significant differences from gel-standard are ^a)^*p* < 0.001 and ^b)^*p* < 0.05.

After 4 hours, the permeable amount of l-theanine from gel-standard was 0.87 mg/cm^2^. In contrast, l-theanine in gel-B and gel-D showed a higher permeability. Permeable amounts of l-Theanine were 2.92 and 3.20 mg/cm^2^, respectively. Although gel-standard and gel-B contained the same stearic acid, stearyl alcohol, and propylene glycol ratio, gel-B, in which all components were dissolved simultaneously, increased the amount of l-theanine permeating the membrane.

l-Theanine with gel-A and gel-C permeated 0.15 and 1.56 mg/cm^2^, respectively (Fig. 2). After 24 hours, membrane permeability of l-theanine in gel-D was 5.71 mg/cm^2^. It follows that l-theanine continued to permeate for a longer period than the other formulations. In contrast, l-theanine in aqueous solution did not permeate the membrane at all after 24 hours (data not shown). Comparing the area under the curve up to 4 hours later, we found that permeability of gel-D was the largest among all formulations at 6.85 mg/cm^2^·h (Table 2).

**Table 2.** Area Under the Curve of l-Theanine in Each Lyogel Through Strat-M Membrane for 4 Hours

	Standard	A	B	C	D
l-Theanine AUC (mg/cm^2^•h)	2.20	0.31	6.08	3.08	6.85

AUC, area under the curve.

### The effect of l-theanine content in the formulation on permeation

**FIG. 3.**
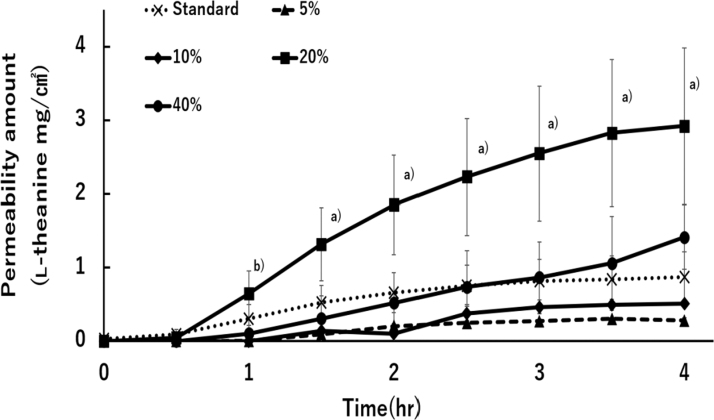
Effects of l-theanine concentration in gel-B through Strat-M membrane permeability. Each point represents mean ± SD (*n* = 3–7). Significant differences from gel-standard are ^a)^*p* < 0.001 and ^b)^*p* < 0.05.

Different amounts of l-theanine were added to gel-B and the amount of permeation compared with l-theanine content. The amount of l-theanine permeation increased in the order of 5%, 10%, 40%, and 20% of l-theanine content. The amount of permeation did not increase with l-theanine content. In other words, the l-theanine content of gel-B did not correlate with the amount of permeation. Furthermore, the amount of l-theanine permeated after 4 hours was approximately twice higher in the 20% l-theanine-containing gel than in the 40% (Fig. 3).

### Lyogel spreadability

A spread meter was used to evaluate the spreadability of lyogels with 20% or 40% l-theanine. In all lyogels, the spreadability was better for the formulation with 20% l-theanine content than for the one with 40% l-theanine content (data not shown).

**FIG. 4.**
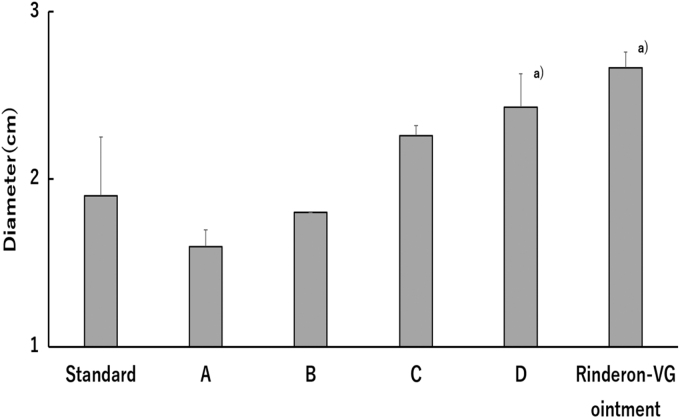
Spreadability of lyogels and Rinderon^®^-VG ointment for 10 seconds. Each column represents mean ± SD (*n* = 6). Significant difference from gel-standard is ^a)^*p* < 0.01.

Gel-D was spread the most when spreadability was compared using formulations containing 20% l-theanine. Gel-A spread 1.6 cm in 10 seconds, whereas gel-D spread 2.4 cm. It was 1.5 times more spreadable than gel-A (Fig. 4). A comparison of the spreadability values of the marketed Rinderon^®^-VG ointment and gel-D showed that they were similar.

### l-Theanine skin permeation by lyogel application

**FIG. 5.**
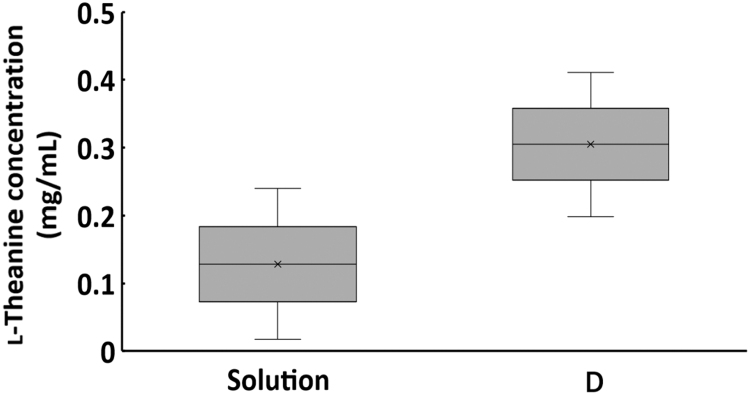
l-Theanine in serum levels of the mice at 24 hours after application. Mice were shaved on the back and samples were applied for 10 days. Blood was collected from their hearts at 24 hours after the last application. (*n* = 3).

Application of gel-D increased l-theanine concentration compared with l-theanine solution in plasma (Fig. 5). The minimum and maximum values for the solution were 0.02 and 0.24 mg/mL, compared with 0.20 and 0.41 mg/mL for gel-D, respectively. The values for gel-D were approximately two times higher than those of the solution when comparing the median value. During the study period, there was no weight loss in any of the mice, and no side effects developed, such as skin change including reddening or rash.

## Discussion

This is a basic study of a lyogel, a preparation applied to the skin, with the aim of obtaining a sustained effect of l-theanine. Although l-theanine has a relaxing effect to alleviate PMS, it has a short blood half-life, and oral administration does not provide the desired effect. To solve this problem, we thought it would be useful to make a transdermal formulation of l-theanine. Absorption of the drug through the skin results in controlled release, allowing blood levels to be maintained for a long time.

There are various types of transdermal formulations, we selected semisolid formulations such as hydrophilic ointment, hydrophobic ointment, cream, oily cream, and lyogel. In the five formulations, l-theanine in lyogel permeated the membrane the most (Fig. 1). The four substrates: plastibase for hydrophobic ointments, macrogol ointment solbase for hydrophilic ointments, hydrophobic cream for cream, and absorptive cream for oily cream, are substrates used to apply drugs to the skin and do not have the property of promoting drug absorption. In contrast, base material of lyogel is characterized by excellent transdermal absorption.^21^

The lyogel formulation was based on the report by Matsushita,^20^ and the lyogel made with this formulation was used as standard. To improve the permeability of l-theanine and the appearance of lyogel application, gel-A, gel-B, and gel-C were prepared based on the standard by examining the ratio of components, or preparation method. First, gel-A, gel-B, and gel-C permeabilities were examined (Fig. 2; Table 2).

Comparing standard and gel-B, which had the same composition ratio but different preparation methods, gel-B had higher l-theanine permeability. Standard was mixed with the other components after dissolving l-theanine in stearyl alcohol. In contrast, gel-B dissolved l-theanine along with all the constituents. l-Theanine was mixed with all the components at the same time, which increased l-theanine's solubility and affected membrane permeability. As a result of comparing the permeabilities using gel-A, gel-B, and gel-C with different propylene glycol ratios, l-theanine in gel-B permeated the Strat-M the most.

This suggests that the propylene glycol ratio affects the permeability. Propylene glycol has been reported to be added to formulations as a cosolvent because of its ability to increase drug permeation through the skin.^22,23^ Each component was mixed with l-theanine, and l-theanine membrane permeation was evaluated. No l-theanine permeated from stearic acid and stearyl alcohol, whereas only propylene glycol allowed l-theanine to permeate the membrane (data not shown). Propylene glycol was also shown to promote l-theanine permeation, suggesting that there is an optimal amount to be added.

In the spreadability test, gel-C had the highest propylene glycol and was the best gel formulation (Fig. 4). It was observed that more propylene glycol tended to have better spreadability and a better appearance when applied on the skin. This suggested that the propylene glycol content affects l-theanine permeability, as well as the physical property of these gels.

The concentration dependence of l-theanine on membrane permeation was evaluated using gel-B. The amount of l-theanine permeation was better in the gel containing 20% l-theanine than 40%, namely, there was no correlation between content and permeation amount of l-theanine (Fig. 3). It was suggested that 40% l-theanine was above the solubility limit in the lyogel base.

Based on the preceding results, this study considered it necessary to develop a lyogel with both excellent membrane permeability and usability. Therefore, gels were prepared without any of the components of lyogel, and the appearance and feel of each gel were evaluated (data not shown). The stearic acid and propylene glycol mixing ratio was fixed at 4.4:296 (mmol), to which 3.7 mmol of stearyl alcohol was added. As a result, the best appearance during application was obtained when stearic acid, stearyl alcohol, and propylene glycol were mixed at 4.4:11.1:296 (mmol), and the gel with this composition was named gel-D.

l-Theanine membrane permeation in gel-D showed similar levels and behavior as in gel-B, and its permeation was sustained for a long time (Fig. 2; Table 2). In addition, gel-D improved the stickiness upon application, which had occurred with gel-A, gel-B, and gel-C (data not shown). Spreadability was also improved, with the best flowability of all the gels, with levels comparable with those of clinically used Rinderon-VG ointment (Fig. 4). As medicines containing l-theanine have not yet been commercialized, we used Rinderon-VG ointment, which is widely used in Japan, as a control to compare the spreading properties. Gel-D was expected, with the necessary permeability, spreadability, and appearance to be a formulation, to have excellent clinical efficacy.

Finally, gel-D was applied to the backs of mice and l-theanine concentration in serum was measured. l-Theanine concentration in serum was higher in gel-D than in l-theanine solution (Fig. 5). The data correlate with the results of the *in vitro* study using Franz cells, which showed that gel-D increases the permeability of l-theanine more than l-theanine solution. l-Theanine gelation strongly suggests the possibility that l-theanine can be absorbed continuously and reaches the systemic circulating bloodstream.

Although it has been shown that l-theanine gelation increases permeability, it is not clear whether the amount permeable is the blood concentration that produces symptomatic relief of PMS. The concentration of l-theanine in the blood required for treatment will be clarified and the optimal number of mice will be examined as data for animal experiments. In the future, the effects on dopamine and serotonin in the brain and toxicity will be investigated by applying theanine-containing lyogel to experimental animals.

The above results indicate that mixing stearic acid, stearyl alcohol, and propylene glycol, the base of lyogel, in optimal proportions and preparing them in an optimal manner will result in a formulation with excellent skin permeability of the l-theanine contained in the product. The composition of lyogel was also studied to obtain a formulation with good application comfort. Although it is suggested that this lyogel-containing l-theanine could be tested in clinical studies to determine whether it is effective for relief of PMS symptoms, lyogel may be suitable as an easy-to-use theanine-containing formulation for women that can relieve PMS symptoms.

## Abbreviations Used

AUC: area under the curve
HPLC: high-performance liquid chromatography
PMDD: premenstrual dysphoric disorder
PMS: premenstrual syndrome
SD: standard deviation
